# Porcine HMGCR Inhibits Porcine Circovirus Type 2 Infection by Directly Interacting with the Viral Proteins

**DOI:** 10.3390/v11060544

**Published:** 2019-06-11

**Authors:** Ting Ouyang, Guyu Niu, Yifang Zhang, Xiaohua Liu, Xinwei Zhang, Shiqi Zhang, Yulu Geng, Daxin Pang, Hongsheng Ouyang, Linzhu Ren

**Affiliations:** 1 Jilin Provincial Key Laboratory of Animal Embryo Engineering, College of Animal Sciences, Jilin University, 5333 Xi’an Road, Changchun 130062, China; ouyt17@mails.jlu.edu.cn (T.O.); niugy9916@mails.jlu.edu.cn (G.N.); lxh18@mails.jlu.edu.cn (X.L.); xwzhang17@mails.jlu.edu.cn (X.Z.); zhangsq9917@jlu.edu.cn (S.Z.); gengyl9917@jlu.edu.cn (Y.G.); pdx@jlu.edu.cn (D.P.); ouyh@jlu.edu.cn (H.O.); 2 College of Animal Medicine, Yunnan Agricultural University, Black Dragon Pool, Kunming 650201, China; zyfkmyn@163.com

**Keywords:** porcine circovirus type 2 (PCV2), 3-hydroxy-3-methylglutaryl coenzyme A reductase (HMGCR), adenosine 5′-monophosphate (AMP)-activated protein kinase (AMPK), protein phosphatase 2 (PP2A), interaction

## Abstract

Porcine circovirus type 2 (PCV2) is the etiological agent of porcine circovirus diseases and porcine circovirus-associated diseases (PCVDs/PCVADs). However, the pathogenesis of PCV2 is not fully understood. We previously found that 3-hydroxy-3-methylglutaryl coenzyme A reductase (HMGCR) is negatively associated with PCV2 infection in vitro and in vivo. HMGCR inhibits the early stages of PCV2 infection, while PCV2 infection induces the phosphorylation of HMGCR to inactivate the protein. In this study, we investigated the possibility that adenosine 5′-monophosphate (AMP)-activated protein kinase (AMPK), and protein phosphatase 2 (PP2A) participate in HMGCR-mediated inhibition of PCV2 infection and the interaction of porcine HMGCR with PCV2 proteins. The results showed that AMPK activity fluctuated in cells during the early stage of PCV2 infection, while PP2A had little effect on PCV2 infection and HMGCR activity. Furthermore, PCV2 infection may enhance or maintain the level of phosphorylated HMGCR by directly interacting with the protein in PK-15 cells. These findings may provide a better understanding of PCV2 pathogenesis, and HMGCR may be a novel PCV2 antiviral target.

## 1. Introduction

Porcine circovirus type 2 (PCV2) is the etiological agent of porcine circovirus diseases and porcine circovirus-associated diseases (PCVDs/PCVADs), which are present in every major swine-producing country in the world [1,2,3]. However, the pathogenesis of PCV2 is not fully understood. PCV2 belongs to the genus *Circovirus* and family *Circoviridae*, the members of which are the smallest nonenveloped, single-stranded, circular DNA viruses [3,4]. Due to its small genomic size (1.7 kb) and highly limited coding capacity, the life cycle of PCV2 relies predominantly on host cell factors [1]. Therefore, finding and identifying host proteins related to PCV2 infection is a promising strategy for elucidating PCV2 pathogenesis and controlling the viral infection.

The enzyme 3-hydroxy-3-methylglutaryl coenzyme A reductase (HMGCR) is the rate-controlling enzyme of the mevalonate pathway, which produces cholesterol and other isoprenoids [5]. Recently, several groups have reported that HMGCR plays important roles in virus infection [6,7,8]. During infection, viruses modulate HMGCR activity to enhance the infection. It was found that multiple Kaposi’s sarcoma-associated herpesvirus (KSHV) viral miRNAs target HMGCR to suppress cholesterol in infected cells during latency, which could potentially be beneficial for viral infection [9]. Dengue virus (DENV) upregulates HMGCR activity by impairing adenosine 5’-monophosphate-activated protein kinase (AMPK) phosphorylation [8]. We previously found that HMGCR is negatively associated with PCV2 infection in vitro and in vivo [10,11,12], as HMGCR inhibits the early stages of PCV2 infection [11], while PCV2 infection induces the phosphorylation of HMGCR to inactivate the protein [12]. However, the mechanism through which HMGCR regulates PCV2 infection has not been elucidated.

It has been reported that HMGCR is regulated by two upstream molecules, AMPK and phosphatase protein phosphatase 2 (PP2A) [8,13]. AMPK inactivates HMGCR via phosphorylation of threonine 172 and serine 872 of HMGCR, while PP2A activates HMGCR directly through its dephosphorylation [8,13]. In this study, we extend our previous findings to examine whether HMGCR regulates PCV2 infection through its upstream molecules or interacts directly with PCV2.

## 2. Materials and Methods

### 2.1. Virus and Cells

Cells were prepared according to the protocol described by Yang et al. [12]. Briefly, PCV-free PK-15 cells were cultured in Dulbecco’s modified Eagle’s medium (DMEM, Gibco, New York, NY, USA) supplemented with 5% fetal bovine serum (FBS, Gibco) and incubated at 37 °C in a 5% CO_2_ atmosphere. Cells were counted using hemocytometer and suspended to the desired dilution.

PCV2 strain CC1 (GenBank accession no. JQ955679) [14] was used in this study. Briefly, PCV-free PK-15 cells at 50% confluence were infected with 10-fold serially diluted PCV2 and cultured in 96-well plates at 37 °C in a 5% CO_2_ atmosphere. Each dilution was replicated eight times. The TCID_50_ values were calculated according to the method described by Yang et al. [12].

### 2.2. Drugs and Antibodies

Lovastatin was purchased from Cayman Chemical (Ann Arbor, MI, USA). Dimethyl sulfoxide (DMSO), FTY720 (PP2A activator), anti-Flag^®^ antibody produced in rabbit, anti-HA antibody produced in rabbit, monoclonal mouse anti-HA antibody and protease inhibitor cocktail were purchased from Sigma (Laramie, WY, USA). Compound C (dorsomorphin, an AMPK inhibitor), metformin (AMPK activator) and the anti-PP2A alpha+beta antibody were all purchased from Abcam (Cambridge, MA, USA). The Anti-PRKAA1+PRKAA2 antibody (phospho-Thr183/Thr172 AMPK), anti-HMG-CoA reductase/HMGCR antibody (C-terminus, FITC), and the anti-PRKAA1+PRKAA2 antibody (AMPK, Internal) were purchased from LifeSpan BioSciences (Seattle, WA, USA). The phospho-PP2A alpha+beta (Tyr307) antibody was purchased from EterLife (London, UK). The HMGCR (phospho-Ser872) antibody was purchased from Biorbyt (Cambridge, MA, USA). The mouse β-actin antibody was purchased from Proteintech (Wuhan, China). The PCV2 capsid antibody was purchased from GeneTex (Irvine, CA, USA). The Cy3-labeled goat anti-mouse IgG (H+L), FITC-labeled goat anti-rabbit IgG (H+L), 4′,6-diamidino-2-phenylindole (DAPI), HRP-conjugated goat anti-rabbit IgG (H+L), HRP-conjugated goat anti-mouse IgG (H+L), BeyoECL Plus Western blot detection system and okadaic acid (PP2A inhibitor) were purchased from Beyotime (Shanghai, China). Mouse anti-HMGCR antibody (C-1, sc-271595) and protein A/G PLUS-agarose beads were purchased from Santa Cruz Biotechnology (Dallas County, TX, USA). Polyclonal rabbit anti-Myc antibody and mouse monoclonal anti-Flag antibody were purchased from Abbkine, Inc. (San Diego, CA, USA). Goat anti-mouse IgG (H+L) was purchased from Jackson ImmunoResearch Inc., (West Grove, PA, USA). Monoclonal mouse Myc-tag antibody was purchased from Proteintech Group, Inc. (Rosemont, IL, USA).

### 2.3. Plasmid Construction

PCV2 DNA was extracted from virus-infected PK-15 cells with the TIANamp Virus DNA/RNA kit (Tiangen, Beijing, China) according to the manufacturer’s protocol. Primers were designed and synthesized according to the Cap gene and Rep gene sequences of PCV2 strain CC1 (GenBank accession no. JQ955679). PCV2 Cap and Rep genes were amplified using the primer pairs Cap-F/Cap-R and Rep-F/Rep-R, respectively. The primers used in this study are described in Table 1. The PCR products were subcloned into Lenti-CAG-WPRE (Hongli biotechnology, Shanghai, China) using an In-Fusion^®^ HD Cloning kit (Takara Bio USA, Inc., Chicago, IL, USA) to generate recombinant expression plasmids pHA-Rep and pMyc-Cap, respectively.

To amplify porcine HMGCR, the primers were designed based on the cDNA sequence of the porcine HMGCR gene (GenBank accession no. DQ432054) using vector NTI 10 (Invitrogen). HMGCR was amplified with primers HMGCR-F/HMGCR-R using pEF-HMG2 [12] as a template. The HMGCR gene was cloned into pCDNA3.1(+) by *Bam*HI and *Eco*RI to generate the expression plasmid pFlag-HMGCR. The plasmids were verified by double enzyme digestion and DNA sequencing conducted by Tiangen Biotech (Beijing, China).

### 2.4. Virus Infection and Treatment

PK-15 cells (10^6^ cells/well) were seeded in 6-well plates for 12 h to reach a confluence of 70–80%. Cells were treated with DMSO, lovastatin, the PP2A activator FTY720, the PP2A inhibitor okadaic acid, the AMPK inhibitor compound C, or the AMPK activator metformin at the indicated concentrations for 1 h. Then, cells were infected with PCV2 strain CC1 at a multiplicity of infection (MOI) of 10 and cultured in fresh medium containing the respective drugs. The proteins and genomic DNA were measured at each indicated time point using western blotting and real-time PCR.

### 2.5. Real-Time PCR

SYBR Green quantitative real-time PCR was performed according to the protocol described by Liu et al. [15]. Briefly, viral genomic DNA was extracted from infected cells using the TIANamp Virus DNA/RNA kit (Tiangen) according to the manufacturer’s protocol. PCR products amplified via the primers PCV2-2A (5′-CACCTTCGGATATACTGTCAA-3′) and PCV2-2B (5′-TACATGGTT ACACGGATATTGTA-3′) [16] were subcloned into pGM-T (Tiangen, China) to generate a standard plasmid pT-PCV2. SYBR Green quantitative real-time PCR was performed with the primer pair PCV2-z1 (5′-TGTAGTATTCAAAGGGCACAGAGC-3′) and PCV2-z2 (5′-CGGATATACTATCAAG CGAACCAC-3′) [17] using the BIO-RAD IQ^TM^5 Multicolor Real-Time PCR Detection System and the Luna^®^ Universal qPCR Master Mix (New England Biolabs, USA). The viral genome copy number was calculated by comparison to the standard curve generated by pT-PCV2 dilution. The experiments were repeated at least three times.

### 2.6. MTS Assay

Cell viability was evaluated according to the protocol described by Yang et al. [12]. Briefly, PK-15 cells were plated in 96-well plates at a density of 5 × 10^3^ cells per well for 24 h. Then, cells were treated with DMSO, the PP2A activator FTY720, the PP2A inhibitor okadaic acid, the AMPK inhibitor compound C, or the AMPK activator metformin at different concentrations for 12 h. Thereafter, the MTS assay was performed using the Cell Counting Kit-8 (Beyotime, Jiangsu, China), and the OD450 values were measured using an ELx800 microplate reader (Bio-TEK) 1 h later. The experiments were repeated at least three times.

### 2.7. Confocal Fluorescence Microscopy

PK-15 cells on coverslips were infected with 200 μL of PCV2 at a MOI of 10 for 1 h [11]. Then, the cells were washed with PBS three times and maintained in fresh medium supplemented with 5% FBS. At 48 h postinfection (hpi), the cells were fixed with 80% cold acetone for 1 h at −20 °C. Thereafter, the cells were washed with PBS three times and incubated with mouse anti-HMGCR antibody for 1 h at 37 °C. After washing with PBS three times, the cells were incubated with the Cy3-labeled goat anti-mouse IgG (H+L, 1:100) secondary antibody for 1 h at 37 °C. Subsequently, the cells were washed with PBS three times and incubated with rabbit anti-Cap antibody (1:100) [18] or rabbit anti-Rep antibody (1:100) [19] previously prepared in our lab for 1 h at 37 °C, followed by incubation with the FITC-labeled goat anti-rabbit IgG (H+L, 1:1000) secondary antibody for 1 h at 37 °C. Finally, the cells were stained with DAPI (1:1000) for 10 min, washed with PBS three times, and the samples were examined under a Zeiss LSM700 confocal microscope (×100).

### 2.8. Coimmunoprecipitation (Co-IP)

PK-15 cells were seeded in a 6-well plate at a density of 1 × 10^6^ cells per well. On the following day, cells were cotransfected with 5 µg of pFlag-HMGCR, pHA-Rep or pMyc-Cap plasmids using Fugene HD Transfection Reagent (Roche) according to the manufacturer’s instructions. Lenti-CAG-WPRE and pCDNA3.1(+) vectors were used as negative controls. Whole-cell extracts were collected 24 h after transfection and washed twice with cold PBS, followed by incubation with RIPA lysis buffer (1000 μL/well) consisting of 50 mM Tris-HCl (pH 8.0), 150 mM NaCl, 1% NP-40, 0.5% sodium deoxycholate, 0.1% sodium dodecyl sulfate (SDS) and complete protease inhibitor cocktail (Roche) for 20 min on ice. After centrifugation for 10 min at 13,000 rpm at 4 °C, supernatants of the whole-cell extracts were collected and divided into two parts. One part was used for coimmunoprecipitation, and the other part was used for immunoblotting analysis.

For coimmunoprecipitation, the supernatants of the whole-cell extracts were incubated with 2 μL of anti-Flag^®^ antibody produced in rabbit, anti-HA antibody produced in rabbit, or polyclonal rabbit anti-Myc antibody at 4 °C overnight on a rocking platform. Thereafter, the supernatants were incubated with 30 μL of protein A/G PLUS-agarose beads for 2 h on a rocking platform, centrifuged at 5000 rpm at 4 °C for 1 min, and then the beads were washed with 500 μL of RIPA lysis buffer three times. Bound proteins were eluted by boiling the beads for 5 min with 2× sample buffer (50 mM Tris-HCl [pH 6.8], 2% SDS, 10% glycerol, and 0.1% bromophenol blue and 1% β-mercaptoethanol). Samples were analyzed by western blot using a monoclonal mouse anti-HA antibody (1:1000), a monoclonal mouse Myc-tag antibody (1:1000) or a mouse monoclonal anti-Flag antibody (1:1000) as the primary antibody. Membranes were then incubated with a goat anti-mouse IgG (H+L) (1:5000) as the secondary antibody and developed using the BeyoECL Plus Western blot detection system according to the manufacturer’s instructions. The whole-cell lysates (input samples) were loaded as input controls for the western blot.

### 2.9. Western Blot (WB)

Total cells were lysed in lysis buffer (25 mM Tris-HCl, pH 7.6, 150 mM NaCl, 1% Triton X-100, 1% sodium deoxycholate, 0.1% SDS, 5% glycerol) with protease inhibitor cocktail added. Whole-cell lysates were separated by SDS-PAGE, electro-transferred to PVDF membranes (Millipore, USA), blocked for 1.5–2 h with 5% skim milk in TBS-T buffer (20 mM Tris-HCl [pH 7.4], 150 mM NaCl, and 0.1% Tween-20), and then incubated with anti-PRKAA1+PRKAA2 (phospho-Thr183/Thr172 AMPK, 1:1000), anti-PRKAA1+PRKAA2 (1:1000, AMPK, internal), anti-phospho-PP2A alpha+beta (Tyr307, 1:1000), anti-PP2A alpha+beta (1:1000), anti-HMGCR (phospho-Ser872, 1:1000), anti-HMG-CoA reductase/HMGCR (1:1000), anti-PCV2 capsid (1:500), or mouse anti-β-actin (1:2000) as the primary antibodies at room temperature for 2 h or at 4 °C overnight. Subsequently, the PVDF membranes were washed with 5% skim milk in TBS-T buffer three times, followed by blotting with HRP-conjugated goat anti-rabbit IgG (H+L, 1:2000) or HRP-conjugated goat anti-mouse IgG (H+L, 1:2000) as the secondary antibody for 1 h, and detected using the BeyoECL Plus Western blot detection system according to the manufacturer’s instructions.

### 2.10. Statistical Analysis

Statistical significance was analyzed using one-way or two-way analysis of variance (ANOVA) with GraphPad Prism software version 5 (GraphPad Software, San Diego, CA, USA), followed by Bonferroni multiple comparison test. The results are expressed as the mean ± standard deviation (SD) of three independent experiments.

## 3. Results

### 3.1. PCV2 Infection Increases Phosphorylation of PP2A, AMPK and HMGCR

**Figure 1:**
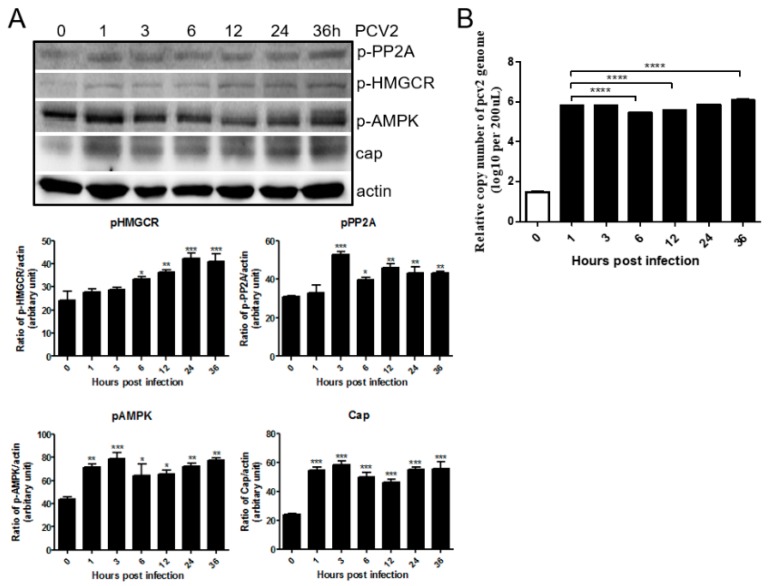
PCV2 infection increases phosphorylation of PP2A, AMPK and HMGCR. Cells were infected with PCV2 and examined using western blot and real-time PCR at the indicated time points. The experiments were repeated at least three times. All data are the means ± standard error (SD) of three independent experiments. *, *p* < 0.05, **, *p* < 0.01, ***, *p* < 0.001, and ****, *p* < 0.0001. (**A**) Western blot analysis and densitometric quantification of the indicated proteins. The graph (lower panel) represents the relative quantification (arbitrary unit) of each protein normalized to β-actin. The bar represents the mean of three independent experiments. (**B**) Real-time PCR of PCV2.

PK-15 cells were infected with PCV2 for 0, 1, 3, 6, 12, 24, and 36 h, and analyzed by western blot and real time PCR. The results showed that phosphorylation of HMGCR increased in a time-dependent manner (Figure 1A). Phosphorylated PP2A and AMPK increased at 1 hpi, but decreased slightly at 3, 6, 12, 24 and 36 hpi, with similar results shown for Cap protein and the genomic copy number of PCV2 (Figure 1). HMGCR is inactivated by AMPK via phosphorylation of threonine 172 and serine 872 of HMGCR, while the phosphatase PP2A activates HMGCR directly through its dephosphorylation [8,11,12,13]. These results indicate that PCV2 infection not only increases the phosphorylation of HMGCR, but also increases the phosphorylation of PP2A and AMPK. Therefore, PCV2 infection enhances AMPK and PP2A activity, but inhibits HMGCR activity, which is consistent with our previous results [11,12]. Therefore, the relationships of PP2A, AMPK and HMGCR during PCV2 infection need to be further evaluated.

### 3.2. Inhibition of HMGCR by Lovastatin Has No Effect on the Activities of AMPK and PP2A during PCV2 Infection

**Figure 2:**
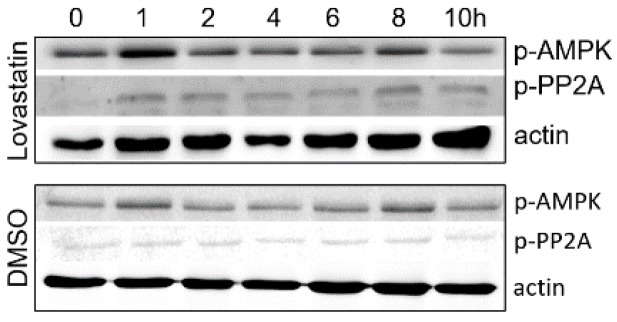
Inhibition of HMGCR by lovastatin has no effect on the activity of AMPK and PP2A during PCV2 infection. Cells were treated with lovastatin (20 μM) or 0.5% DMSO and infected with PCV2, then analyzed by western blot at the indicated time points. The experiments were repeated at least three times.

Statins, including lovastatin and atorvastatin, are common inhibitors of HMGCR [10,12]. Previously, we confirmed that 20 μM lovastatin or 0.5% DMSO had no cytopathic effects on PK-15 cells [11,12]. To evaluate the levels of AMPK and PP2A, cells were cultured in DMEM containing 20 μM lovastatin or 0.5% DMSO, followed by PCV2 infection. No significant difference in the level phosphorylated PP2A was observed in both lovastatin- and DMSO-treated cells during PCV2 infection (Figure 2). In addition, the levels of phosphorylated AMPK increased at 1 and 8 hpi, but decreased to normal levels in both lovastatin- and DMSO-treated cells during PCV2 infection at 2, 4, 6 and 10 hpi (Figure 2), suggesting that AMPK activity fluctuated during the early stage of PCV2 infection. Moreover, these results also suggest that inhibition of HMGCR by lovastatin has no effect on the activity of AMPK and PP2A during PCV2 infection.

### 3.3. PP2A Has Little Effect on PCV2 Infection and HMGCR Activity

**Figure 3:**
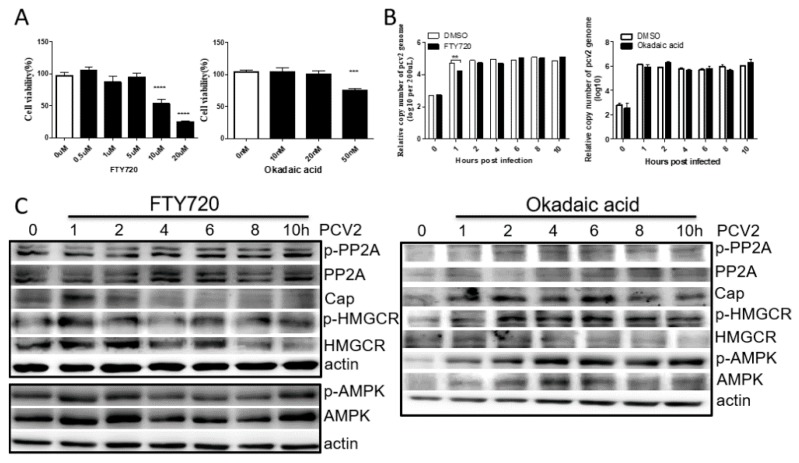
PP2A has little effect on PCV2 infection and HMGCR activity. Cells were treated with FTY720 (PP2A activator) or okadaic acid (PP2A inhibitor) and infected with PCV2, and analyzed by western blot and real-time PCR at the indicated time points. The results are expressed as the mean ± SD of three independent experiments. The experiments were repeated at least three times. **, *p* < 0.01, ***, *p* < 0.001, and ****, *p* < 0.0001. (**A**) Cytopathic effects of drugs. (**B**) Real-time PCR. (**C**) Western blot.

To evaluate the cytopathic effect of FTY720 (PP2A activator) or okadaic acid (PP2A inhibitor), cells were cultured in DMEM containing different concentrations of FTY720 or okadaic acid and examined using the Cell Counting Kit-8 according to the manufacturer’s instructions. The results showed that cell viability was significantly decreased in DMEM containing 10 μM and 20 μM FTY720, as well as DMEM containing 50 nM okadaic acid (Figure 3A). Therefore, 5 μM FTY720 and 10 nM okadaic acid was used in the following study.

Cells were incubated with FTY720 or okadaic acid, followed by PCV2 infection. As shown in Figure 3B, the copy number of PCV2 was significantly decreased in FTY720-treated cells compared with that of DMSO-treated cells at 1 hpi, while the level of PCV2 Cap protein was increased at 1 hpi and significantly decreased later (Figure 3C). When PP2A was inhibited with okadaic acid, no significant difference in the copy number and Cap protein of PCV2 was observed between the okadaic acid-treated cells and DMSO-treated cells (Figure 3B,C). These results indicate that activated PP2A can inhibit PCV2 infection, which mainly targets the transcriptional or translational level of the viral infection.

Moreover, it has been reported that AMPK activity can be inhibited by activated PP2A [20,21,22]. Thus, the levels of AMPK phosphorylation were also examined in FTY720-, okadaic acid- or DMSO-treated cells during PCV2 infection. The results showed that the AMPK activities increased at 1 and 10 hpi and decreased at the other times, which is consistent with the results shown in Figure 1 and Figure 2, suggesting that PP2A has no effect on AMPK activity during PCV2 infection (Figure 3B,C). These results further confirm that AMPK activity fluctuated during the early stage of PCV2 infection. Furthermore, as shown in Figure 3C, the levels of phosphorylated HMGCR changed in a manner similar to that of phosphorylated AMPK, suggesting that HMGCR is regulated by AMPK during PCV2 infection.

### 3.4. HMGCR Activity Is Mainly Regulated by AMPK during PCV2 Infection

**Figure 4:**
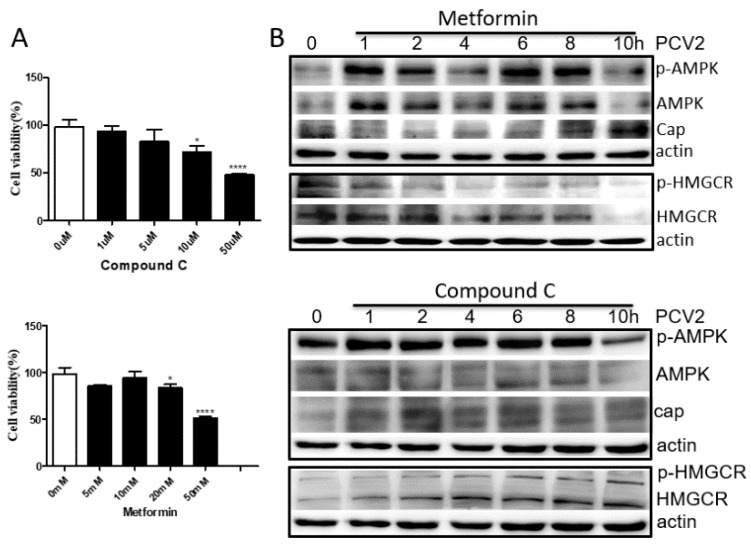
HMGCR activity is mainly regulated by AMPK during PCV2 infection. Cells were treated with compound C (AMPK inhibitor) or metformin (AMPK activator) and infected with PCV2, and analyzed by western blot at the indicated time points. The results are expressed as the mean ± SD of three independent experiments. The experiments were repeated at least three times. *, *p* < 0.05, ***, *p* < 0.001, and ****, *p* < 0.0001. (**A**) Cytopathic effects of drugs. (**B**) Western blot.

To select a suitable concentration of compound C (AMPK inhibitor) or metformin (AMPK activator), cells were cultured in DMEM containing different concentrations of compound C or metformin and examined using the Cell Counting Kit-8. The results showed that cell viability was significantly decreased in DMEM containing 10 μM compound C and 20 mM metformin (Figure 4A). Therefore, 5 μM compound C and 10 mM metformin were used in the following study.

When cells treated with compound C or metformin were infected with PCV2, the levels of phosphorylated AMPK increased at 1–2 hpi and 6–8 hpi, but decreased at 4 and 10 hpi (Figure 4B), further confirming that AMPK activity fluctuated during the early stage of PCV2 infection. Furthermore, the level of phosphorylated HMGCR increased in compound C-treated cells and decreased in the metformin-treated cells in a time-dependent manner during PCV2 infection (Figure 4B). These results suggest that HMGCR activity is mainly regulated by AMPK, while PCV2 infection may enhance or maintain the level of phosphorylated HMGCR. Thus, whether HMGCR directly interacts with PCV2 needs to be evaluated.

### 3.5. Construction of Recombinant Expression Plasmids

**Figure 5:**
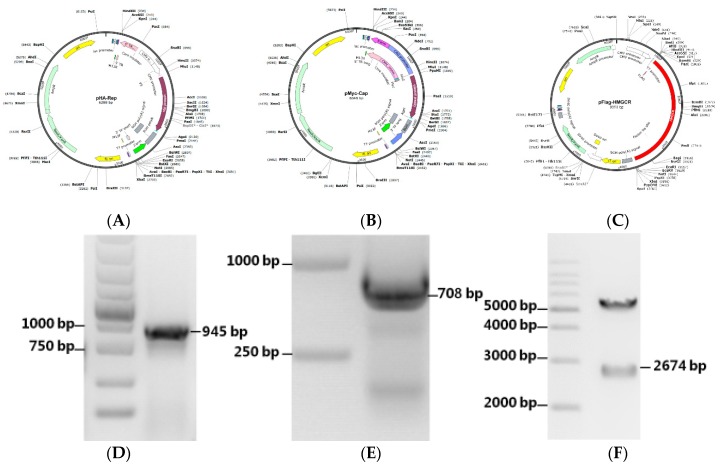
Construction scheme of the plasmids used in this study. The Cap and Rep genes of PCV2 were cloned into Lenti-CAG-WPRE by *Mlu*I and *Age*I to generate recombinant expression plasmids pHA-Rep (**A**,**D**) and pMyc-Cap (**B**,**E**), respectively and were further verified by PCR. The porcine HMGCR gene was cloned into pCDNA3.1(+) by *Bam*H I and *Eco*R I to generate the expression plasmid pFlag-HMGCR (**C**) and confirmed by double digestion with *Bam*H I and *Eco*R I (**F**).

PCV2 Cap and Rep genes were amplified and subcloned into Lenti-CAG-WPRE to generate the recombinant expression plasmids pHA-Rep (Figure 5A) and pMyc-Cap (Figure 5B), respectively. HMGCR was amplified and cloned into pCDNA3.1(+) by *Bam*H I and *Eco*R I to generate the expression plasmid pFlag-HMGCR (Figure 5C). The plasmids were verified by PCR (Figure 5D,E) or enzyme digestion (Figure 5F) and DNA sequencing (data not shown).

### 3.6. HMGCR Interacts with the Cap Protein of PCV2

**Figure 6:**
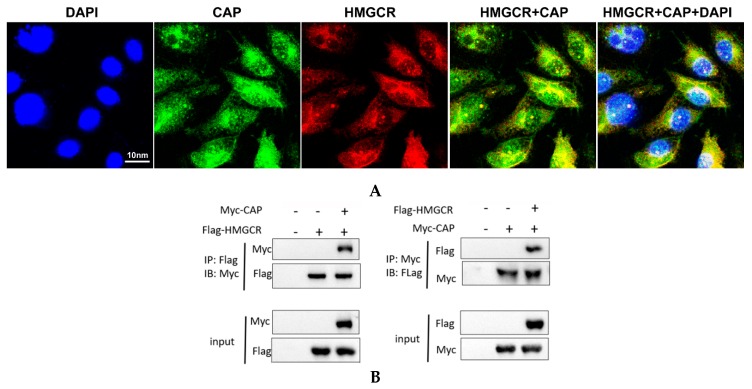
Interaction between HMGCR and PCV2 Cap protein. (**A**) Colocalization of HMGCR and PCV2 Cap protein. HMGCR (Cy3; red), PCV2 Cap protein (FITC; green), DAPI (blue) and overlap (yellow), scale bar: 10 nm. (**B**) Co-IP of HMGCR and PCV2 Cap protein. Flag (HMGCR), Myc (Cap).

The Cap protein of PCV2 contains the main antigenic determinant of the virus, and the protein also plays an important role in the initiation of PCV2 DNA replication [3,23]. To examine whether the negative effect of HMGCR on the replication of PCV2 is associated with the viral Cap protein, we investigated the interaction and colocalization of HMGCR with the Cap protein during PCV2 infection using an immunofluorescence assay. We observed that there was a significant colocalization of HMGCR and the viral Cap protein (Figure 6A). The majority of the HMGCR protein accumulated together with the Cap protein in the cytoplasm and around the nucleus of the virus-infected cells.

To further investigate the interaction between PCV2 Cap and HMGCR, an immunoprecipitation experiment was conducted. The results showed that the Myc-labeled protein coimmunoprecipitated with the anti-Flag^®^ antibody produced in rabbit (Figure 6B, left panel), and the Flag-fused protein was detected in immunoprecipitates obtained with the polyclonal rabbit anti-Myc antibody (Myc-Cap) at 24 h posttransfection (Figure 6B, right panel), indicating that the Myc-labeled protein interacts with the Flag-fused protein. Since HMGCR was fused to a Flag tag and the viral Cap protein was labeled with a Myc tag in the present study, these results demonstrated that HMGCR interacts with Cap protein during PCV2 infection.

### 3.7. HMGCR Interacts with the Rep Protein of PCV2

**Figure 7:**
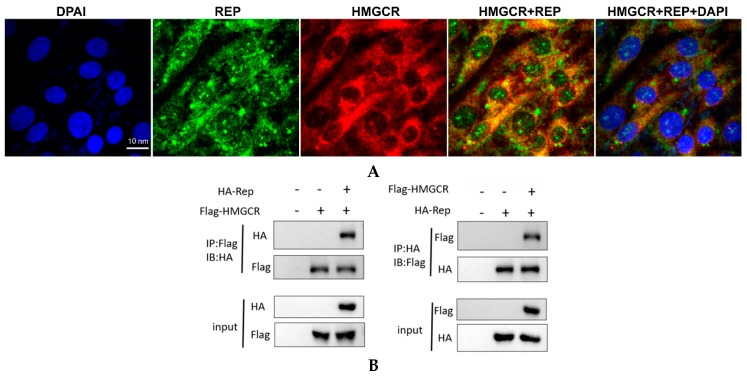
Interaction between HMGCR and PCV2 Rep protein. (**A**) Colocalization of HMGCR and PCV2 Rep protein. HMGCR (Cy3; red), PCV2 Rep protein (FITC; green), DAPI (blue) and overlap (yellow), scale bar: 10 nm. (**B**) Co-IP of HMGCR and PCV2 Rep protein. Flag (HMGCR), HA (Rep).

The Rep protein of PCV2 is a viral DNA replication-associated protein that is highly conserved and encoded by the ORF1 gene of the virus [3]. It has been reported that the Rep protein not only interacts with cellular filament protein and transcriptional regulator c-Myc, but also interacts with Cap protein [24]. Therefore, we analyzed the subcellular localization of HMGCR and the Rep protein. HMGCR was stained using a mouse anti-HMGCR antibody followed by incubation with a Cy3-labeled goat anti-mouse IgG (H+L) secondary antibody, while the Rep protein was stained using a rabbit anti-Rep antibody and a FITC-labeled goat anti-rabbit IgG. Confocal microscopy revealed that the porcine HMGCR and the viral Rep protein displayed high degrees of colocalization within the cytoplasm and perinuclear region of the virus-infected cells (Figure 7A).

To further investigate the interaction between PCV2 Rep and HMGCR, an immunoprecipitation experiment was performed. The results showed that the anti-Flag^®^ antibody produced in rabbit immunoprecipitated not only the Flag-tagged protein, but also the HA-tagged protein after 24 h of transfection (Figure 7B, left panel) and vice versa (Figure 7B, right panel), indicating that the HA-labeled protein can interact with the Flag-tagged protein. Since HMGCR was fused to a Flag tag and the viral Rep protein was labeled with an HA tag in the present study, these results demonstrate that HMGCR interacts with the Rep protein in the cytoplasm during PCV2 infection, suggesting a role for HMGCR in PCV2 replication.

## 4. Discussion

We previously found that HMGCR is negatively associated with PCV2 infection in vitro and in vivo [10,11,12]. HMGCR inhibits the early stages of PCV2 infection [11], while PCV2 infection induces the phosphorylation of HMGCR to inactivate the protein [12]. In this study, we also found that PCV2 infection enhanced the activity of AMPK and PP2A, two upstream molecules involved in HMGCR phosphorylation (Figure 1). To evaluate whether AMPK and PP2A participate in HMGCR inhibition during PCV2 infection, PK-15 cells were treated with drugs targeting AMPK or PP2A, and infected with PCV2. The results showed that AMPK activity fluctuated in cells during the early stage of PCV2 infection and this fluctuation had no relationship with the activator/inhibitor treatments (Figure 2, Figure 3 and Figure 4). Furthermore, PP2A had little effect on PCV2 infection and HMGCR activity (Figure 3), and HMGCR activity was mainly regulated by AMPK, while PCV2 infection may enhance or maintain the level of phosphorylated HMGCR (Figure 4). Therefore, we hypothesized that HMGCR may interact with PCV2.

Subsequently, we investigated the interaction and colocalization of porcine HMGCR with Cap and Rep proteins of PCV2 using immunofluorescence and coimmunoprecipitation assays. We found that the PCV2 Cap/Rep proteins and HMGCR were significantly colocalized at 48 hpi, which indicates that HMGCR affects PCV2 infection via interactions with the viral proteins. It has been reported that PCV2 is closely associated with mitochondria [25], and the viral Cap protein recruits the host dynein/microtubule machinery to travel through the cytoplasm towards the nuclear membrane [26]. Other groups found that PCV2 Cap proteins are expressed in the cytoplasm, transported to the nucleus by a nuclear localization signal (NLS) in the flexible arginine-rich motif (ARM) domain of Cap (which is recognized by host receptors of the importin family and other cofactors), and then exported to the cytoplasm through the phosphorylation of the NLS [26,27]. As expected, the majority of the HMGCR protein accumulated together with the viral Cap or Rep proteins in the cytoplasm of the virus-infected cells. These results suggest that the interactions between the viral proteins and host proteins occur mainly in the cytoplasm, which was also confirmed by coimmunoprecipitation assays (Figure 6 and Figure 7).

HMGCR is a transmembrane enzyme containing eight transmembrane domains that localize to both the membrane of the endoplasmic reticulum (ER) and peroxisomes [5,28]. The N-terminal domain of HMGCR is composed of 339 amino acids and is integrated into membranes by virtue of eight membrane-spanning segments, while the 548-amino acid C-terminal reductase domain projects into the cytosol and exerts all of the enzymatic activity [29]. Interestingly, significant colocalization of the Cap protein and HMGCR was observed in the perinuclear region of the virus-infected cells. It has been reported that filamentous vimentin protein has a restrictive effect on the replication of PCV2 in PK-15 cells by colocalizing with the viral Cap protein around the nucleus and forming a special structure [30]. Ravi and colleagues found that HMGCR mediates changes in F-actin structure [6]. Therefore, one possible reason for this result is that HMGCR may interact with filament proteins or actin or microtubules during PCV2 infection, which enables HMGCR to localize around the nucleus. Furthermore, since the mouse anti-HMGCR antibody (C-1, sc-271595) used in this study was raised against amino acids 589-888 mapping to the C-terminus of HMGCR, we hypothesized that another possible reason for this finding was that interactions between HMGCR and the viral protein(s) resulted in HMGCR accumulating around the nucleus. However, the mechanism needs to be further elucidated. Moreover, truncated mutation constructs of HMGCR, Rep and Cap proteins were constructed, and studies on the interactions between different domains of HMGCR and the viral proteins are currently ongoing in our lab.

## 5. Conclusions

In conclusion, this study investigated the possibility that AMPK and PP2A participate in HMGCR-inhibited PCV2 infection, as well as the interactions between porcine HMGCR and PCV2 proteins. The results showed that AMPK activity fluctuates in cells during the early stage of PCV2 infection, while PP2A has little effect on PCV2 infection and HMGCR activity. Furthermore, PCV2 infection may enhance or maintain the level of phosphorylated HMGCR by directly interacting with the protein in PK-15 cells. These findings may provide a better understanding of PCV2 pathogenesis, and HMGCR may be a novel PCV2 antiviral target.

## Figures and Tables

**Table 1:** Primers used in this study.

Primer	Oligonucleotide Sequences (5′–3′)	Cloning Site	Size (bp)
Rep-F	acgcgtatgtacccatacgacgtaccagattacgctcccagcaaaaagaatggaagaag	*Mlu* I *Age* I	945
Rep-R	cgaccggttcagtaatttatttcatatggaaattcag		
Cap-F	acgcgtatggagcagaagctgatctcagaggaggacctgacgtatccaaggaggcgtta	*Mlu* I *Age* I	708
Cap-R	cgaccggtttaagggttaagtggggggtctt		
HMGCR-F	ggatccatgttgtcaagactcttccgaatgc	*Bam*H I *Eco*R I	2674
HMGCR-R	gaattctcaagctgccttcttagtgcaag		

Note: Restriction sites are underlined.
